# Thrombocytopenia as an important determinant of poor prognosis in patients with pyogenic liver abscess: a retrospective case series

**DOI:** 10.3389/fsurg.2023.1192523

**Published:** 2023-07-25

**Authors:** Sheng-zhong Li, Shao-hua Liu, Meng Hao, Tian Yu, Song Hu, Li Liu, Zhe-long Liu

**Affiliations:** ^1^ Department of Surgery, Wuhan Jinyintan Hospital, Tongji Medical College, Huazhong University of Science and Technology, Wuhan, China; ^2^ Department of Endocrinology, Tongji Hospital, Tongji Medical College, Huazhong University of Science and Technology, Wuhan, China; ^3^ Branch of National Clinical Research Center for Metabolic Diseases, Wuhan, China; ^4^ Department of Gastroenterology, Zigui County People’s Hospital, Yichang, China; ^5^ Department of Gastroenterology, Tongji Hospital, Tongji Medical College, Huazhong University of Science and Technology, Wuhan, China

**Keywords:** pyogenic liver abscess, thrombocytopenia, platelet count, prognosis, cross-sectional analysis

## Abstract

**Background:**

Thrombocytopenia and poor prognosis in severe conditions are associated. However, the clinical significance of thrombocytopenia in pyogenic liver abscess (PLA) has not been evaluated.

**Objective:**

To evaluate the association between thrombocytopenia and the prognosis of patients with PLA.

**Methods:**

A consecutive case series of 458 adult patients with PLA hospitalized at Tongji Hospital (Wuhan, China) between October 2011 and June 2021 was included in this cross-sectional analysis. Patient data were compared between the thrombocytopenia and non-thrombocytopenia groups. Multivariate logistic regression, receiver operating characteristic (ROC) curve and propensity score -matched analyses (PSM) were performed.

**Results:**

Of the 458 patients with PLA, 94 (20.5%) developed thrombocytopenia, 19 (4.1%) developed septic shock, 14 (3.1%) were admitted to the ICU, and 15 (3.3%) died during hospitalization. Thrombocytopenia was independently associated with shock (95%CI = 3.529–57.944, *P* < 0.001), ICU admission (95%CI = 1.286–25.733, *P* = 0.022), and mortality (95%CI = 1.947–34.223, *P* = 0.004) in multivariate regression analysis. ROC analysis showed that thrombocytopenia may be an identified marker of shock [area under the ROC curve (AUC), 0.8119; cut-off, 92.50; *P* < 0.0001], ICU admission (AUC, 0.7484; cut-off, 82.50; *P* < 0.0015), and mortality (AUC, 0.7827; cut-off, 122.50; *P* < 0.002). These findings remained consistent across 86 pairs of patients analyzed for PSM analyses.

**Conclusions:**

Thrombocytopenia is an independent risk factor for poor prognosis in PLA and patients may be more prone to adverse outcomes.

## 1. Introduction

Pyogenic liver abscess (PLA) is an infectious disease caused by a microbial infection that leads to liver necrosis, septic shock, and other serious consequences (1). The disease has an acute onset and complex condition, and missing the best treatment time may lead to various complications that seriously harm the health and quality of life of PLA patients (2). The incidence of PLA is higher in Asian countries, which can reach 12–18 cases per 100,000 people per year, with a mortality rate of approximately 2%–31% (3, 4).Therefore, prognostic markers should be identified to provide more aggressive and timely resuscitation for patients and plan effective treatment for patients with PLA to improve prognosis.

Thrombocytopenia is the most common hemostatic disorder in critically ill patients, with a prevalence of approximately 40%–53% (5). Previous studies have reported that platelet counts (PLT) regulate inflammation by controlling tissue integrity, and protecting against infection (6). Hence, PLT is considered to be valuable in predicting some disease outcomes, such as sepsis (7), COVID-19 (8, 9), and cancer (10). Zhou (11) demonstrated that patients with severe thrombocytopenia had more blood transfusions, more days requiring advanced support, and worse outcomes than those in the normal group. Orak et al. (12). retrospectively studied 330 patients diagnosed with sepsis in the emergency department and found that the PLTs were lower in patients who died than in the survivors. What's more, platelets have been used as prognostic markers for other hepatobiliary duct diseases, such as cholecystitis/cholangitis, which are closely associated with PLA (13–15). PLA is an inflammatory disease, and we hypothesized that thrombocytopenia may be also associated with its prognosis, which is worthy of our in-depth and detailed exploration and research.

To date, many studies have demonstrated a number of risk factors related to the prognosis of liver abscesses, such as malnutrition, pleural effusion, fever, multiple organ dysfunction syndrome (MODS), presence of gas formation, the size of abscess, and microbiology (16–20). However, few studies have explored the direct association between thrombocytopenia and PLA prognosis. If PLTs, as a common laboratory indicator that is easy to detect, could help to recognize patients with PLA at high-risk for adverse outcomes, it would have even greater clinical benefits. This study was conducted to explore the relationship between thrombocytopenia and PLA prognosis to provide a new and convenient prognostic marker of PLA.

## 2. Methods

### 2.1. Study design and participant

This was a retrospective collection of a consecutive case series of 458 patients with PLA admitted to Tongji Hospital, Tongji Medical College, Huazhong University of Science and Technology, between October 2011 and June 2021.

Patients with PLA were identified by retrospectively searching the medical record code (search term “pyogenic liver abscess”, ICD 10 code = K750) in the medical record department. The inclusion criteria for analysis were as follows: (1) diagnosis of PLA according to the following diagnostic criteria: a. imaging examination found liver abscess lesion (either magnetic resonance imaging (MRI), computed tomography (CT), or ultrasound (US); b. the patient had fever, chills, liver percussion pain, and other clinical manifestations and signs; c. positive bacterial culture; d. the lesions subsided after antibiotic treatment; e. liver biopsy or surgical pathology confirmed; a was essential for diagnosis, and b-e were non-essential diagnostic criteria; (2) age ≥18 years; (3) complete key laboratory results and imaging data. The following were the exclusion criteria: (1) incomplete clinical data; (2) amebic liver abscess or parasitic liver abscess; and (3) underlying conditions at risk for thrombocytopenia (hematologic malignancies, chemotherapy, cirrhosis, and chronic heart failure). The management of PLA was in accordance to the *Expert consensus on multidisciplinary management of intra-abdominal infections* (21) and *Quality Improvement Guidelines for Percutaneous Drainage/Aspiration of Abscess and Fluid Collections* (22). Typically, the duration of parenteral antibiotic treatment of liver abscesses is between 4 and 6 weeks. Interval imaging was typically performed after a specific period of antibiotic treatment. The interval between imaging studies also varied depending on the patient's clinical progress, response to therapy, and the physician's judgment.

The study was approved by the ethics committee of Tongji Hospital of Huazhong University of Science and Technology and conducted in accordance with the Declaration of Helsinki (TJ-IRB20221240). This study was exempt from informed consent because of its retrospective design.

### 2.2. Data collection

Data collected from the electronic medical record system in hospital included patients' demographic characteristics (age and sex), comorbidities (diabetes mellitus, hypertension, chronic respiratory disease, cardiovascular disease, gastrointestinal and hepatobiliary diseases, and malignancy), clinical symptoms and signs (fever, nausea and vomiting, abdominal distension or pain, diarrhea, fatigue), vital signs on admission (temperature, heart rate, respiration rate, and blood pressure), lab tests upon admission [blood routine test, liver function, renal function, serum levels of inflammatory markers, coagulation function, serum lipid parameters, random blood glucose, troponin I, n-terminal pro-brain natriuretic peptide(NT-proBNP), and other related indexes], bacterial cultures, treatments, imaging findings, serious complications, and adverse outcomes including septic shock, acute renal failure, acute hepatic failure, heart failure, myocardial infarction, pulmonary edema, pulmonary infection, acute respiratory distress syndrome (ARDS), pleural effusion, ICU occupancy, and death.

### 2.3. Definitions

Thrombocytopenia was defined as a PLT less than 125 × 10^9^/L (laboratory reference range 125–350 × 10^9^/L). Patients with liver abscess were categorized into the thrombocytopenia and non-thrombocytopenia groups based on PLTs less than or above 125 × 10^9^/L, respectively. Septic shock was defined as acute circulatory failure with uncorrectable hypotension unexplained by other causes, despite sufficient fluid resuscitation (23). Acute renal failure was diagnosed when serum creatinine above 176 µmol/L, or an absolute increase was greater than 44 µmol/L (24). Acute hepatic injury was defined by WHO diagnostic criteria as alanine aminotransferase (ALT), aspartate aminotransferase (AST), total bilirubin (TBIL) where any one of these markers was more than 1.25 times the reference value upper limit. Heart failure was defined in accordance with the guidelines of the Heart Failure Association of the European Society of Cardiology (25). Myocardial infarction was defined as a serum level of hypersensitive cardiac troponin I (hs-cTnI) >34 pg/ml (26). Pulmonary edema is mainly diagnosed on the basis of pulmonary imaging findings. ARDS was defined according to The Berlin Definition of Acute Respiratory Distress Syndrome (2012) (27).

### 2.4. Statistical analysis

**Figure 1:**
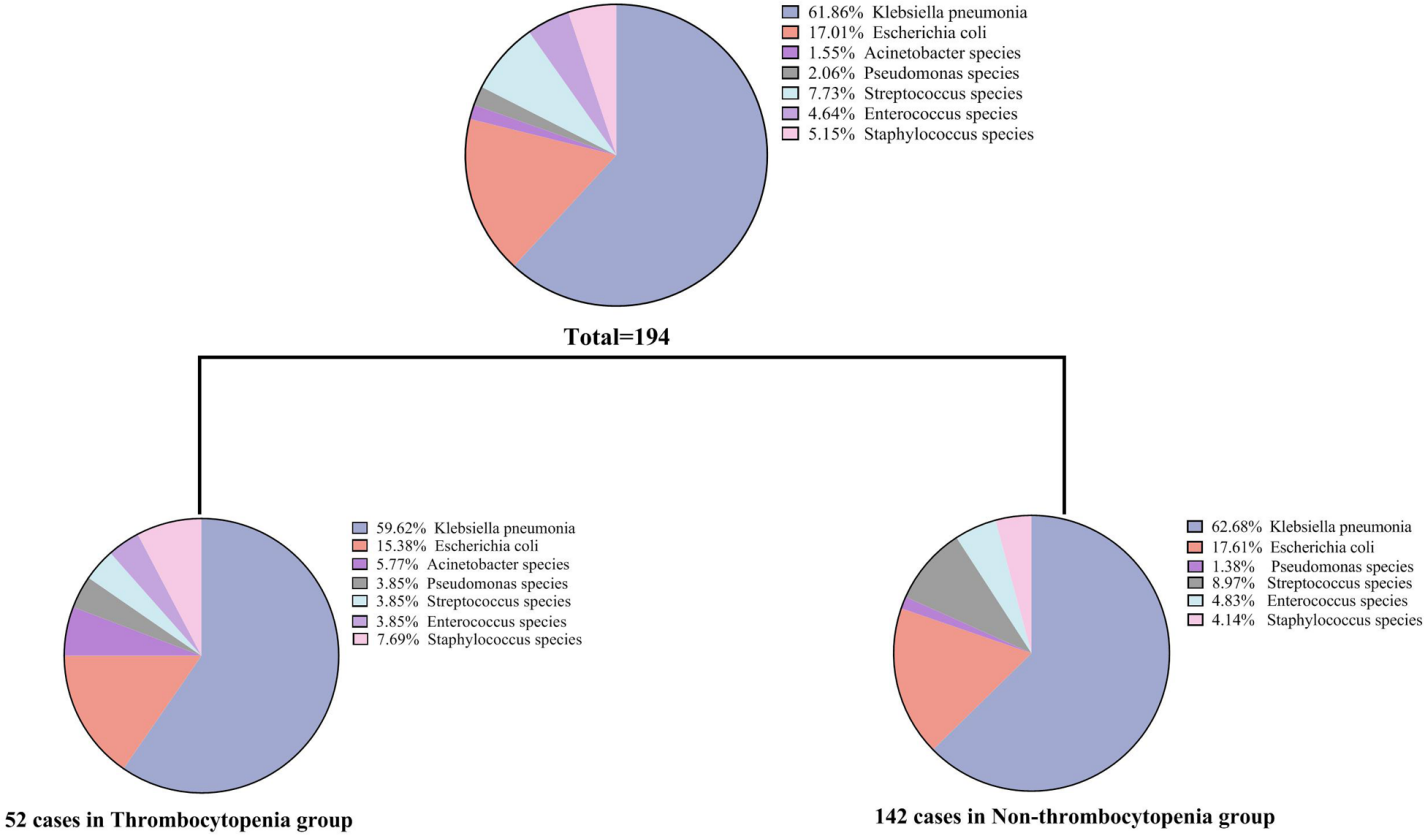
Profiles of isolated microorganisms in patients with pyogenic liver abscess.

The mean ± standard deviation was used to express the continuous variable of normal distribution, and the median (quartile distance) was used to express the continuous variable of skewness distribution. Categorical variables were expressed as frequencies and percentages (%). For comparison between the two groups (thrombocytopenia group vs. non-thrombocytopenia group), continuous variables were analyzed by Student's *t*-test or Mann-Whitney U test, and categorical variables were analyzed by Fisher's exact or chi-squared test. Univariate logistic regression analysis was performed to ascertain the possible risk factors that showed a relationship with shock, ICU admission, and mortality. Variables without collinearity were selected for the multivariable analysis to assess the association between thrombocytopenia and PLA, taking into consideration previous research findings (28), clinical implications, and significant variables identified in the univariate logistic regression analysis. Finally, age, gender, hemoglobin (Hb), TBIL, creatinine, white blood cell, ARDS, presence of gas and pleural effusion were included in the multivariate analysis. The distribution of isolated microorganisms of the total or two groups (thrombocytopenia group vs. non-thrombocytopenia group) is presented in Figure 1. Receiver operating characteristic curve (ROC) was plotted to evaluate the discriminatory performance for adverse outcomes (shock, ICU admission, and mortality) of PLA according to the value of the area under the ROC curve (AUC). To adjust for additional confounding factors, a propensity score matching analysis was performed. Standardized mean differences and *t*-tests were used to ensure baseline demographics were comparable between the two groups. After matching, the standardized mean differencece of all matching factors in the *t*-test was not statistically significant. The matched variables included: age, sex, fever, nausea and vomiting, abdominal distension or pain, diarrhea, fatigue and muscle pain, palpitation, cough and sputum, dizziness or headache, SBP, DBP, heartrates, diabetes mellitus, hypertension, cardiovascular disease, malignancy, liver and gallbladder stones, viral hepatitis, fatty liver disease, presence of gas, hemoglobin, total bilirubin, direct bilirubin, ALT, AST, albumin, creatinine. SPSS version 20.0 software (SPSS Inc, Chicago, IL, USA) and GraphPad Prism (ver.9, GraphPad Software, La Jolla, USA) were used for all statistical analyses and figure construction. Statistical significance was defined as a two-sided *P*-value <0.05.

## 3. Results

### 3.1. Demographics and baseline characteristics of patients with PLA

Table 1 shows the demographic data of patients at admission. A total of 458 patients with liver abscess were enrolled in this study, with an average age of 53.0 ± 0.6 years, of whom 131 (28.6%) patients were female. The most common clinical symptom was abdominal distension or abdominal pain (*n* = 230, 50.2%), followed by fatigue and muscle pain (*n* = 178, 38.9%) and fever (*n* = 129, 28.2%). Less common symptoms included dyspnea (*n* = 8, 1.7%) and disturbance of consciousness (*n* = 7, 1.5%). Diabetes mellitus (*n* = 101, 22.1%) was the most common comorbidity, followed by liver or gallbladder stones (*n* = 100, 21.8%) and hypertension (*n* = 78, 17.0%).

**Table 1:** Demographics and baseline characteristics between thrombocytopenia group and non-thrombocytopenia group with pyogenic liver abscess.

Characteristics	Overall population				PSM population		
	Total (*n* = 458)	Thrombocytopenia group (*n* = 94)	Non-thrombocytopenia group (*n* = 364)	*P-*value	Thrombocytopenia group (*n* = 86)	Non-thrombocytopenia group (*n* = 86)	*P*-value
Age (years)	53.0 ± 0.6	54.7 ± 1.3	52.5 ± 0.7	0.154	54.87 ± 12.29	53.86 ± 12.88	0.599
Female, *n* (%)	131 (28.6)	24 (25.5)	107 (29.7)	0.460	66 (76.7)	67 (77.9)	0.856
Clinical symptoms/signs							
Fever, *n* (%)	129 (28.2)	27 (28.7)	102 (28.0)	0.893	24 (27.9)	29 (33.7)	0.409
Nausea and vomiting, *n* (%)	83 (18.1)	24 (25.5)	59 (16.2)	**0.036**	24 (27.9)	22 (25.6)	0.730
Abdominal distension or pain, *n* (%)	230 (50.2)	44 (46.8)	186 (51.1)	0.458	41 (47.7)	36 (41.9)	0.443
Diarrhea, *n* (%)	36 (7.9)	10 (10.6)	26 (7.1)	0.262	10 (11.6)	8 (9.3)	0.618
Fatigue and muscle pain, *n* (%)	178 (38.9)	47 (50.0)	131 (36.0)	**0.013**	44 (51.2)	43 (50.0)	0.879
Chest pain, *n* (%)	18 (3.9)	3 (3.2)	15 (4.1)	0.679	3 (3.5)	6 (7.0)	0.496
Palpitation, *n* (%)	27 (5.9)	10 (10.6)	17 (4.7)	**0.029**	9 (10.5)	8 (9.3)	0.798
Cough and sputum, *n* (%)	43 (9.4)	11 (11.7)	32 (8.8)	0.388	11 (12.8)	11 (12.8)	1.000
Dizziness or headache, *n* (%)	49 (10.7)	15 (16.0)	34 (9.3)	0.064	13 (15.1)	15 (17.4)	0.680
Dyspnea, *n* (%)	8 (1.7)	5 (5.3)	3 (0.8)	**0.011**	2 (2.3)	3 (3.5)	1.000
Disturbance of consciousness, *n* (%)	7 (1.5)	3 (3.2)	4 (1.1)	0.156	2 (2.3)	1 (1.2)	1.000
Co-morbidity							
Diabetes mellitus, *n* (%)	101 (22.1)	26 (27.7)	75 (20.6)	0.141	23 (26.7)	28 (32.6)	0.404
Hypertension, *n* (%)	78 (17.0)	16 (17.0)	62 (17.0)	0.998	16 (18.6)	15 (17.4)	0.843
Cardiovascular disease, *n* (%)	13 (2.8)	4 (4.3)	9 (2.5)	0.353	4 (4.7)	5 (5.8)	1.000
Chronic respiratory disease, *n* (%)	22 (4.8)	6 (6.4)	16 (4.4)	0.422	5 (5.8)	7 (8.1)	0.549
Urologic diseases, *n* (%)	8 (1.7)	2 (2.1)	6 (1.6)	0.752	2 (2.3)	2 (2.3)	1.000
Malignancy, *n* (%)	22 (4.8)	4 (4.3)	18 (3.9)	0.780	4 (4.7)	5 (5.8)	1.000
Liver and gallbladder stones, *n* (%)	100 (21.8)	22 (23.4)	78 (21.4)	0.679	21 (24.4)	19 (22.1)	0.718
Viral hepatitis, *n* (%)	54 (11.8)	16 (17.0)	38 (10.4)	0.078	14 (16.3)	13 (15.1)	0.834
Fatty liver disease, *n* (%)	49 (10.7)	12 (12.8)	37 (10.2)	0.467	10 (11.6)	10 (11.6)	1.000
Vital sign at admission							
Temperature, ◦C	36.7 (36.4, 37.2)	36.8 (36.5, 37.4)	36.7 (36.4, 37.2)	0.509	36.5 (36.5, 37.5)	36.8 (36.5, 38.0)	0.301
SBP, mmHg	120 ± 0.9	115 ± 2.3	122 ± 0.9	**0.002**	115 ± 20	117 ± 17	0.521
DBP, mmHg	75 ± 0.5	72 ± 1.3	76 ± 0.6	**0.001**	71 ± 13	72 ± 10	0.497
Heart rates, /min	86 (78, 98)	91 (80, 103)	86 (78, 96)	**0.012**	91 (80, 103)	91 (80, 106)	0.604
Respiratory rates, /min	20 (20, 20)	20 (20, 20)	20 (20, 20)	0.900	20 (20, 20)	20 (20, 20)	0.254
The size of abscess (≥5 cm)	49 (10.7)	9 (9.6)	40 (11.0)	0.452	8 (9.3)	7 (8.1)	0.556
Presence of gas	39 (8.5)	10 (10.6)	29 (8.0)	0.408	9 (10.5)	9 (10.5)	1.000
Multiloculation	84 (18.3)	22 (23.4)	62 (17.0)	0.238	21 (24.4)	22 (25.6)	0.706

Bold value means statistically significant (*P* < 0.05). Data are presented as mean ± SE or median (interquartile range) for continuous variables and *n* (%) for categorical variables. *P*-values comparing thrombocytopenia group and non-thrombocytopenia group are from Student's *t*-test, Mann–Whitney *U*-test, *χ*^2^ test, or Fisher's exact test. We had data on the size of the abscess for 72 patients, while data on multiloculation were available for 405 patients in this study.

PSM, propensity score-matched; SBP, systolic blood pressure; DBP, diastole blood pressure.

Patients were categorized, based on PLT, into either the thrombocytopenia group (*n* = 94) or non-thrombocytopenia group (*n* = 364). Compared with the non-thrombocytopenia group, the thrombocytopenia group was more likely to report nausea, vomiting, fatigue, muscle pain, palpitations, and dyspnea and had lower blood pressure and higher heart rates for vital signs at admission (*P* < 0.05). However, no significant differences in size of abscess, presence of gas, or multiloculation were observed between the two groups.

### 3.2. Baseline laboratory parameters of patients with PLA

The thrombocytopenia group showed significantly decreased levels of lymphocyte, albumin, total cholesterol, high density lipoprotein-cholesterol (HDL-C), low density lipoprotein-cholesterol (LDL-C), and fibrinogen, as well as increased levels of inflammatory markers like C-reactive protein, hepatic function indicators (TBIL, direct bilirubin, ALT, and AST), kidney function indicators (urea nitrogen and creatinine), coagulation function indicators (PT and D-D dimer), cardiac parameters (NT-proBNP and cTnI), and others, such as random blood glucose, compared with the non-thrombocytopenia group (Table 2, *P* < 0.05).

**Table 2:** Laboratory indices between thrombocytopenia and non-thrombocytopenia group with pyogenic liver abscess.

Laboratory data	Normal range	Overall population				PSM population		
		Total (*n* = 458)	Thrombocytopenia group (*n* = 94)	Non-Thrombocytopenia group (*n* = 364)	*P*-value	Thrombocytopenia group (*n* = 86)	Non-Thrombocytopenia group (*n* = 86)	*P*-value
White blood cell, 10^9^/L	3.5–9.5	10.1 (7.0, 13.8)	9.4 (5.9, 13.4)	10.4 (7.4, 13.7)	0.085	9.03 (5.49, 13.34)	11.96 (8.21, 14.93)	**0.004**
Neutrophil count, 10^9^/L	1.8–6.3	7.9 (5.1, 11.5)	7.8 (4.2, 12.2)	7.9 (5.2, 12.3)	0.663	7.30 (4.08, 11.74)	9.88 (6.48, 13.25)	**0.008**
Lymphocyte count, 10^9^/L	1.10–3.2	1.2 (0.8, 1.6)	0.8 (0.6, 1.1)	1.4 (1.0, 1.8)	**<0.001**	0.83 (0.59, 1.15)	0.94 (0.68, 1.44)	**0.020**
Hemoglobin, g/L	115–150	115 (101, 129)	110 (97, 124)	116 (102, 130)	0.175	111 ± 23	110 ± 22	0.815
Platelet count, 10^9^/L	125–350	232 (141, 321)	79 (47, 99)	268 (196, 349)	**<0.001**	82 (47, 99)	238 (165, 337)	**<0.001**
CRP, mg/L	<1	115 (59, 190)	143 (100, 200)	97 (51, 184)	**0.003**	162 ± 91	137 ± 78	0.161
PCT, ng/ml	<0.05	1.0 (0.2, 7.8)	8.3 (2.5, 24.2)	0.5 (0.1, 2.4)	**0.000**	8.25 (2.53, 23.9)	1.55 (0.21, 13.58)	**0.015**
Total bilirubin, µmol/L	<=21	11.8 (7.9, 18.2)	17.2 (12.0, 42.3)	10.9 (7.5, 16.4)	<0.001	17 (12, 41)	13 (9,24)	**0.003**
Direct bilirubin, µmol/L	<=8	5.4 (3.1, 9.4)	10.0 (5.8, 27.4)	4.7 (2.8, 7.4)	**<0.001**	10 (6, 26)	7 (4, 14)	**0.002**
ALT, U/L	<33	31 (18, 55)	47 (31, 113)	28 (17, 48)	**<0.001**	47 (31, 93)	43 (21, 86)	**0.015**
AST, U/L	<32	26 (18, 45)	49 (23, 98)	24 (17, 38)	**<0.001**	46 (23, 93)	39 (21, 68)	0.185
ALP,U/L	135–214	140 (98, 210)	153 (113, 224)	137 (96, 204)	0.125	154 (112, 214)	195 (120,	**0.038**
*γ*-GT, U/L	6–42	115 (63, 193)	131 (69, 205)	112 (59, 191)	0.220	134 (76, 207)	137 (91, 285)	0.257
Albumin, g/L	35–52	31.7 ± 0.3	28.4 ± 0.7	32.5 ± 0.3	**<0.001**	29 ± 7	29 ± 6	0.592
TC, mmol/L	<5.8	3.1 (2.4, 3.8)	2.6 (2.1, 3.5)	3.2 (2.6, 3.9)	**<0.001**	2.7 (2.1, 3.6)	3.0 (2.3, 3.6)	0.102
TG, mmol/L	<1.7	1.2 (0.9, 1.9)	1.4 (1.0, 2.1)	1.2 (0.8, 1.7)	0.067	1.4 (1.0, 2.2)	1.4 (1.0, 1.9)	0.820
PT, s	11.5–14.5	14.7 (13.9, 15.7)	15.1 (14.2, 16.8)	14.6 (13.9, 15.5)	**<0.001**	15 (14, 17)	15 (14, 16)	0.253
APTT, s	29–42	41.0 (37.1, 45.0)	41.0 (36.4, 45.2)	41.1 (37.1, 44.9)	0.863	41 ± 7	42 ± 6	0.842
Random blood glucose, mmol/L	-	7.1 (5.6, 10.9)	8.6 (6.5, 13.1)	6.9 (5.5, 10.3)	**0.003**	8.6 (6.4, 12.9)	9.0 (6.0, 13.1)	0.857
BUN, mmol/L	2.6–7.5	4.4 (3.2, 5.9)	6.4 (3.8, 9.9)	4.1 (3.1, 5.3)	**<0.001**	99.7 ± 5.5	98.5 ± 6.5	0.165
Creatinine, µmol/L	59–104	67 (54, 83)	77 (59, 103)	65 (54, 79)	**<0.001**	76 (58, 99)	69 (60, 84)	**0.232**
NT-proBNP, pg/ml	<62.9	665 (296, 2,353)	1,449 (671, 3,094)	391 (145, 1,001)	**<0.001**	1,301 (665, 2,596)	430 (98, 695)	**0.007**
cTnI, pg/ml	<=34.2	1.8 (0.0, 7.6)	2.3 (0.0, 16.2)	0.1 (0.0, 4.0)	**0.049**	3.05 (0.02, 16.20)	0.25 (0.00, 5.70)	0.022

Bold value means statistically significant (*P* < 0.05). Data are presented as median (interquartile range) for continuous variables. *P*-values comparing thrombocytopenia and non-thrombocytopenia group are from Mann–Whitney *U*-test.

PSM, propensity score-matched**;** CRP, C-reactive protein; PCT, procalcitonin; ALT, alanine aminotransferase; AST, aspartate aminotransferase, ALP, alkaline phosphatase; γ-GT, γ-glutamate transpeptida; TC, Total cholesterol; TG, Triglyceride; HDL-C, high-density lipoprotein cholesterol; LDL-C, low-density lipoprotein cholesterol; PT, prothrombin time; APTT, activated partial thromboplastin time; Fib, Fibrinogen; BUN, blood urea nitrogen; NT-proBNP, n-terminal pro-brain natriuretic peptide; cTnI, cardiac troponin I.

### 3.3. Profiles of isolated microorganisms of patients with PLA

Of the 458 patients with PLA, 194 (42.4%) showed positive bacterial culture results. Twenty-six (12.37%) of the cases were multiple bacterial infections. Among them, 22 cases were cultured with two kinds of bacteria, and 4 cases were cultured with three or more kinds of bacteria. Additionally, there were 4 cases of bacterial infection with fungal infection. As shown in Figure 1, *Klebsiella pneumoniae* was the most common pathogenic microorganism in both the thrombocytopenia and non-thrombocytopenia groups, accounting for 61.86% of all patients, followed by *Escherichia coli* (17.01%). *Streptococcus* (7.73%), *Staphylococcus* (5.15%), and *Enterococcus* (4.64%) accounted for a certain proportion. There was no difference in the bacterial culture composition ratio between the thrombocytopenia and non-thrombocytopenia groups (*P* = 0.318).

### 3.4. Treatment and clinical outcomes of patients with PLA

As shown in Table 3, 128 patients (27.9%) received conservative treatment with antibiotics alone, 278 patients (60.7%) received antibiotics with abscess puncture and drainage, and 33 patients (7.2%) received antibiotics with surgery. In these areas, there were no statistical differences between the thrombocytopenia and non-thrombocytopenia groups regarding the treatment received. Albumin infusion (*P* = 0.002), glucocorticoid use (*P* < 0.001), and antiviral drug use (*P* = 0.010) were significantly higher in the patients with thrombocytopenia than in those without thrombocytopenia.

**Table 3:** Treatment and clinical outcomes between thrombocytopenia and non-thrombocytopenia group with pyogenic liver abscess.

	Overall population				PSM population		
	Total	Thrombocytopenia group (*n* = 94)	Non-Thrombocytopenia group (*n* = 364)	*P*-value	Thrombocytopenia group (*n* = 86)	Non-Thrombocytopenia group (*n* = 86)	*P*-value
Treatments							
Antibiotics alone	128 (27.9)	29 (30.9)	99 (27.2)	0.482	26 (30.2)	25 (29.1)	0.867
Antibiotics plus percutaneous drainage	278 (60.7)	59 (62.8)	219 (60.2)	0.645	53 (61.6)	53 (61.6)	1.000
Antibiotics plus surgical	33 (7.2)	5 (5.3)	28 (7.7)	0.428	5 (5.8)	4 (4.7)	1.000
Albumin infusion	202 (44.3)	57 (60.6)	145 (40.1)	**0.002**	52 (60.5)	45 (52.3)	0.282
Antiviral drug	29 (6.3)	12 (12.8)	17 (4.7)	**0.010**	12 (14.0)	7 (8.1)	0.224
Serious complications							
Septic shock	19 (4.1)	15 (16.0)	4 (1.1)	**<0.001**	12 (14.0)	2 (2.3)	**0.010**
Acute renal injury	28 (6.2)	13 (14.0)	15 (4.2)	**<0.001**	10 (11.6)	6 (7.0)	0.294
Acute hepatic injury	212 (46.3)	68 (72.3)	144 (39.6)	**<0.001**	63 (73.3)	52 (60.5)	0.075
Heart failure	30 (6.6)	17 (18.1)	13 (3.6)	**<0.001**	14 (16.2)	3 (3.5)	**0.009**
Myocardial infarction	13 (2.8)	11 (11.7)	2 (0.5)	**0.001**	8 (9.3)	0 (0.0)	**0.007**
Pulmonary edema	12 (2.6)	8 (8.5)	4 (1.1)	**0.001**	6 (7.0)	1 (1.2)	0.117
ARDS	10 (2.2)	7 (7.4)	3 (0.8)	**<0.001**	6 (7.0)	1 (1.2)	0.117
Pleural effusion	187 (40.8)	56 (59.6)	131 (36.0)	**<0.001**	51 (59.3)	38 (44.2)	**0.047**
ICU admission	14 (3.1)	9 (9.6)	5 (1.4)	**<0.001**	7 (8.1)	3 (3.5)	0.329
Mortality	15 (3.3)	11 (11.7)	4 (1.1)	**<0.001**	10 (11.6)	0 (0.0)	**0.001**

Bold value means statistically significant (*P* < 0.05). Data are presented as *n* (%) for categorical variables. *P*-values comparing thrombocytopenia and non-thrombocytopenia group are from *χ*^2^ test or Fisher's exact test.

PSM, propensity score-matched; ARDS, acute respiratory distress syndrome.

Compared with the non-thrombocytopenia group, patients with thrombocytopenia were more likely to develop serious complications, such as septic shock (*P* < 0.001), acute renal injury (*P* < 0.001), acute hepatic injury (*P* < 0.001), heart failure (*P* < 0.001), myocardial infarction (*P* = 0.001), pulmonary edema (*P* = 0.001), ARDS (*P* < 0.001), and pleural effusion (*P* < 0.001). In addition, ICU occupancy (*P* < 0.001) and mortality rates (*P* < 0.001) were significantly higher in the thrombocytopenia group than in the non-thrombocytopenia group.

Furthermore, we compared baseline data of liver abscess patients grouped by death, ICU admission, and shock, and the results are presented in the Supplementary (Tables S1–S3).

### 3.5. Independent association between thrombocytopenia and poor prognosis in patients with PLA

Based on univariate analysis (Table 4), decreased hemoglobin (*P* = 0.003), PLT (*P* = 0.001), and albumin (*P* < 0.001), as well as pleural effusion (*P* = 0.018) and ARDS (*P* < 0.001) were correlated with septic shock; female sex, decreased hemoglobin (*P* = 0.011), PLT (*P* = 0.003), albumin (*P* < 0.001), increased ALT (*P* = 0.020), and pleural effusion (*P* = 0.004) and ARDS (*P* < 0.001) were correlated with ICU admission; and decreased hemoglobin (*P* = 0.009), PLT (*P* = 0.003), albumin (*P* < 0.001),and increased TBIL, creatinine (*P* = 0.011) and ARDS (*P* < 0.001)were correlated with mortality in PLA patients. Moreover, the independent association between thrombocytopenia and poor prognosis was determined using a multivariate logistic regression analysis. After adjusting for age, sex, hemoglobin, albumin, ALT/TB, and pleural effusion, thrombocytopenia remained independently associated with shock, ICU admission, and mortality in patients with PLA. As seen in Table 5, in model 3, the ORs for thrombocytopenia (PLT < 125 × 10^9^/L) were 14.300 (95% CI = 3.529–57.944; *P* < 0.001), 5.753 (95% CI = 1.286–25.733; *P* = 0.022), and 8.163 (95% CI = 1.947–34.223; *P* = 0.004) for shock, ICU admission, and mortality, respectively. Additionally, when the PLT was tested as a continuous variable, with each 10-unit increase in PLT, the adjusted ORs of shock, ICU admission, and mortality were 0.936 (95% CI = 0.890–0.984; *P* = 0.010), 0.932 (95% CI = 0.875–0.992; *P* = 0.027), and 0.915 (95% CI = 0.852–0.982; *P* = 0.014), respectively.

**Table 4:** Univariate logistic regression analysis for risk factors associated with shock, ICU admission and mortality in patients with pyogenic liver abscess.

	Overall population						PSM population	
	Shock		ICU admission		Mortality		Adverse outcomes	
	Odd ratio (95% CI)	*P*	Odd ratio (95% CI)	*P*	Odd ratio (95% CI)	*P*	Odd ratio (95% CI)	*P*
Age	0.990 (0.958,1.024)	0.574	0.984 (0.947,1.022)	0.402	1.011 (0.971,1.051)	0.601	0.985 (0.950, 1.022)	0.422
Male/Female	1.526 (0.497,4.688)	0.460	0.287 (0.098,0.845)	**0.023**	0.795 (0.266,2.372	0.681	0.495 (1.183, 1.344)	0.168
Hemoglobin, g/L	0.969 (0.949,0.989)	**0.003**	0.969 (0.946,0.993)	**0.011**	0.969 (0.947,0.992)	**0.009**	0.977 (0.857, 0.998)	**0.032**
Fever (Yes vs. No)	1.516 (0.583,3.940)	0.393	1.957 (0.666,5.756)	0.222	0.2.302 (0.817,6.485)	0.115	0.957 (0.347, 2.645)	0.933
White blood cell, 10^9^/L	1.073 (1.015, 1.135)	**0.014**	1.056 (0.987, 1.130)	0.113	1.042 (0.972, 1.117)	0.248	0,994 (0.924, 1.069)	0.864
Lymphocyte count, 10^9^/L	0.788 (0.390, 1.592)	0.506	0.997 (0.578, 1.718)	0.991	0.869 (0.645, 1.173)	0.359	1.166 (0.789, 1.725)	0.441
Platelet count, 10^9^/L	0.991 (0.986,0.996)	**0.001**	0.991 (0.985,0.997)	**0.003**	0.989 (0.983,0.995)	**<0.001**	0.992 (90.985, 0.998)	**0.011**
ALT, U/L	1.001 (0.999, 1.004)	0.152	1.003 (1.000, 1.006)	**0.020**	0.997 (0.987, 1.008)	0.629	0.997 (0.990, 1.004)	0.475
PT, s	1.025 (0.957, 1.097)	0.480	1.038 (0.976, 1.103)	0.233	1.160 (0.880, 1.513)	0.292	1.174 (0.940, 1.467)	0.158
Albumin, g/L	0.823 (0.753, 0.899)	**<0.001**	0.787 (0.703, 0.880)	**<0.001**	0.879 (0.804, 0.961)	**0.004**	0.897 (0.821, 0.980)	**0.016**
Total bilirubin, µmol/L	1.008 (0.999, 1.017)	0.098	1.006 (0.994, 1.018)	0.348	1.012 (1.003, 1.021)	**0.008**	0.999 (0.987, 1.011)	0.847
Creatinine, µmol/L	1.004 (1.000,1.009)	0.059	1.004 (0.999, 1.009)	0.157	1.005 (1.001, 1.010)	**0.011**	0.998 (0.989, 1.007)	0.678
CRP, mg/L	1.000 (0.994, 1.006)	0.996	1.005 (0.998, 1.012)	0.201	1.001 (0.993, 1.010)	0.792	0.999 (0.992, 1.006)	0.758
PCT, ng/ml	1.001 (0.996, 1.006)	0.708	1.000 (0.992, 1.008)	0.963	0.998 (0.977, 1.020)	0.891	1.005 (0.969, 1.043)	0.783
NT-proBNP, pg/ml	1.000 (1.000, 1.000)	0.046	1.000 (1.000, 1.000)	0.296	1.000 (1.000, 1.000)	0.676	1.000 (1.000, 1.000)	0.915
cTnI, pg/ml	1.004 (0.996, 1.012)	0.351	1.000 (0.998, 1.002)	0.890	1.000 (0.997, 1.002)	0.895	1.006 (0.998, 1.014)	0.152
Diabetes mellitus	1.276 (0.448, 3.632)	0.648	1.431 (0.439, 4.662)	0.552	1.807 (0.603, 5.413)	0.290	0.757 (0.283, 2.023)	0.578
Cardiovascular disease	4.578 (0.940, 22.284)	0.060	2.769 (0.335, 22.916)	0.345	2.565 (0.312, 21.124)	0.381	4.294 (0.983, 18.753)	0.053
Hypertension	0.261 (0.034, 1.986)	0.195	2.000 (0.611, 6.548)	0.252	0.743 (0.164, 3.360)	0.700	0.782 (0.214, 2.851)	0.709
Malignancy	2.465 (0.532, 11.409)	0.249	<0.001 (<0.001, -)	0.998	3.254 (0.687, 15.404)	0.137	2.302 (0.444, 11.937)	0.321
Liver and gallbladder stones	1.293 (0.454, 3.681)	0.630	1.450 (0.445, 4.725)	0.538	1.314 (0.409, 4.220)	0.646	1.487 (0.531, 4.165)	0.450
Viral hepatitis	0.876 (0.197, 3.899)	0.862	2.102 (0.567, 7.785)	0.266	0.526 (0.068, 4.079)	0.538	0.564 (0.123, 2.587)	0.462
Pleural effusion	3.300 (1.231, 8.846)	**0.018**	9.223 (2.039, 41.708)	**0.004**	2.233 (0.781, 6.384)	0.134	3.162 (1.095, 9.136)	**0.033**
ARDS	84.778 (19.510, 368.380)	**<0.001**	82.500 (19.439, 350.134)	**<0.001**	26.485 (6.534, 107.348)	**<0.001**	64.714 (7.267, 576.314)	**<0.001**
The size of abscess (<5 cm)	0.219 (0.019, 2.547)	0.225	–	–	–	–	–	–
Presence of gas	2.099 (0.584, 7.545)	0.256	3.091 (0.825, 11.586)	0.094	4.239 (1.283, 14.008)	**0.018**	0.944 (0.200, 4.45)	0.942
Multiloculation	1.096 (0.351, 3.422)	0.874	0.687 (0.149, 3.162)	0.630	1.773 (0.520, 5.773)	0.370	1.235 (0.437, 3.488)	0.690

Bold value means statistically significant (*P* < 0.05). “Adverse oucomes” included shock, ICU admission, and death.

PSM, propensity score-matched; CI: confidence interval; ALT, alamine aminotransferase; PT, prothrombin time; CRP, C-reactive protein; PCT, procalcitonin; ARDS, acute respiratory distress syndrome.

**Table 5:** Association of thrombocytopenia with shock, ICU admission and mortality in patients with pyogenic liver abscess.

Thrombocytopenia	Model 1		Model 2		Model 3	
	OR (95% CI)	*P*	OR (95% CI)	*P*	OR (95% CI)	*P*
Shock						
PLT < 125	17.089 (5.523,52.874)	<0.001	18.220 (5.801,57.225)	<0.001	14.300 (3.529, 57.944)	<0.001
Per 10 unit increase	0.915 (0.870, 0.963)	0.001	0.914 (0.869, 0.961)	<0.001	0.936 (0.890, 0.984)	0.010
ICU admission						
PLT < 125	7.602 (2.484, 33.265)	<0.001	8.996 (2.854, 28.359)	<0.001	5.753 (1.286, 25.733)	0.022
Per 10 unit increase	0.913 (0.860, 0.969)	0.003	0.909 (0.857, 0.963)	0.001	0.932 (0.875, 0.992)	0.027
Mortality						
PLT < 125	11.928 (3.706,38.392)	<0.001	12.078 (3.733, 39.081)	<0.001	8.163 (1.947, 34.223)	0.004
Per 10 unit increase	0.894 (0.840, 0.952)	<0.001	0.894 (0.840, 0.952)	<0.001	0.915 (0.852, 0.982)	0.014
Adverse outcomes^(PSM population)^						
PLT < 125	4.686 (1.497, 14.668)	0.008	4.742 (1.504, 14.947)	0.008	4.221 (1.148, 15.526)	0.030
Per 10 unit increase	0.920 (0.862, 0.981)	0.011	0.921 (0.864, 0.982)	0.012	0.923 (0.859, 0.991)	0.027

Model 1: unadjusted; Model 2: adjusted for age and gender; Model 3: adjusted for age, gender, Hb, TBIL, creatinine, White blood cell, ARDS, presence of gas and pleural effusion. “Adverse oucomes” included shock, ICU admission, and death.

PSM, propensity score-matched; PLT, platelet count; Hb, hemoglobin; TBIL, total bilirubin; ARDS, acute respiratory distress syndrome.

### 3.6. Propensity score-matched analysis

The PSM analysis resulted in a total of 86 pairs of study participants. Due to the limited number of subjects, several analyses were constrained. Therefore, we combined three outcome events (shock, ICU admission, and death) as “adverse outcomes” for analysis. It is noteworthy that the baseline features were well balanced between the two groups, as indicated in Table 1. Consistent with findings in the overall population, the PSM analysis demonstrated an independent association between thrombocytopenia and adverse outcomes in patients with PLA.

### 3.7. Prognostic value of PLT

**Figure 2:**
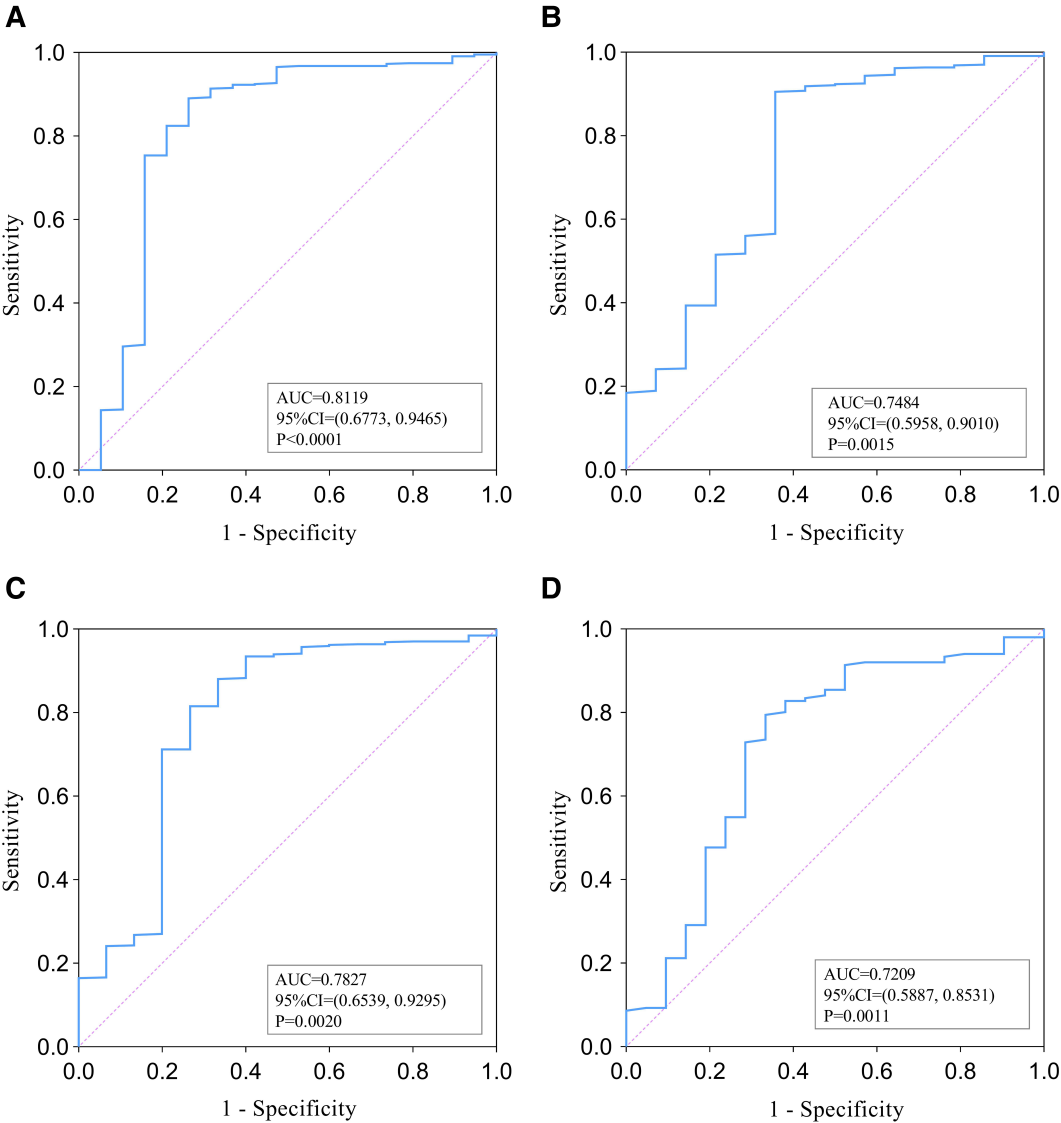
ROC analysis of PLT for the identification of shock (**A**), ICU admission (**B**), and mortality (**C**) among overall papulation with pyogenic liver abscess. (**D**) presents ROC analysis of PLT's ability to identify adverse outcomes in PSM populations.

As shown in Figure 2, ROC analysis was performed to evaluate the relationship between PLT and PLA prognosis. For the discriminative ability of shock in patients with PLA, the AUC value of the PLT was 0.8119 (95% CI = 0.6773–0.9465, *P* < 0.0001) (Figure 2A). The optimal cut-off (the value of PLT when the Youden index reaches the maximum) was 92.5 × 10^9^/L) with a corresponding sensitivity and specificity of 89.07% and 73.68%, respectively. The AUC value of PLT as an identified marker of ICU admission was 0.7484 (95% CI = 0.5958–0.9010, *P* = 0.0015) (Figure 2B), and the cut-off value was 82.50 × 10^9^/L with a corresponding sensitivity and specificity of 90.54% and 64.29%, respectively. The AUC of PLT for the discriminative ability of mortality was 0.7827 (95% CI = 0.6539–0.9295, *P* = 0.002) (Figure 2C) and the cut-off value was 122.50 × 10^9^/L with a corresponding sensitivity and specificity of 81.53% and 73.33%, respectively. In addition, in the PSM analysis, the AUC value of PLT was 0.7209 (95%CI = 0.5887–0.8531, *P* = 0.0011) as an indicator of adverse outcomes in PLA patients (Figure 2D). The cut-off value was 82.5 × 109/L, the sensitivity was 79.47%, and the specificity was 66.67%.

## 4. Discussion

In the study involving 458 patients with PLA, thrombocytopenia was found in 20% of cases. Patients with thrombocytopenia were more likely to have deranged (including inflammatory markers and hepatic and renal function indicators were deranged), increased risk of complications, higher ICU admission rates, and elevated mortality. ROC analysis indicated that PLT, an inexpensive and easily obtained biomarker, may be a good candidate to meaningfully identify the poor prognosis of PLA.

Platelets are small anucleate circulating cells that are increasingly recognized as key effector cells that modulate host responses to inflammation and infections (29, 30). Platelets play a key role in innate immunity by interacting with other immune cells, through multiple receptors on their surface, such as Toll-like receptor 4 (31). Thrombocytopenia is a common complication of infection-related diseases. Platelets can interact with pathogens through a variety of platelet damage-related molecular pattern receptors, with the most common causative agent being viruses (28%), followed by bacteria (28%), and fungi (15%) (31). Current studies indicate that the causes of infection or sepsis-associated thrombocytopenia are complex, not fully elucidated, and may involve several factors, for example, a decrease in platelet production. The liver is the most vital site for the synthesis of thrombopoietin (TPO), a crucial platelet-stimulating factor that modulates platelet production (31). Liver injury due to PLA may result in an absolute or relative deficiency in TPO levels in these patients, leading to thrombocytopenia. In addition, various inflammatory cytokines can cause thrombocytopenia by destroying stem cells in the bone marrow (32, 33). This is followed by increased platelet destruction and consumption. Infections can stimulate platelet activation and aggregation through various pathways leading to thrombocytopenia (34, 35). Bacteria, such as Escherichia coli, Staphylococcus aureus, and Streptococcus pneumonia, can directly stimulate platelet activation and platelet-leukocyte aggregation by binding to and activating platelet receptors such as toll-like receptors or by participating plasma proteins related pathways (36). The release of diverse antimicrobial peptides can give rise to cell destruction and tissue damage. Endothelial cell injury and its release of inflammatory factors can also induce platelet activation and aggregation, increase platelet consumption or thrombosis (31, 37–39), and lead to thrombocytopenia. Studies have shown that the release or upregulation of sialidase during infection leads to the hydrolysis of sialic acid, a natural sugar acid that protects platelets from destruction (40–42), thereby causing thrombocytopenia. Additionally, treatment-related drug induction has been shown to cause thrombocytopenia. For example, vancomycin (43), linezolid, cephalosporin, and chloroquine phosphate (44, 45) can produce antiplatelet antibodies that induce increased platelet destruction. Finally, thrombocytopenia was found to be associated with a lower protein concentration and higher fluid balance, which may suggest a hemodilution effect caused by heavy fluid rehydration during treatment (46).

The pathophysiological mechanism of adverse outcomes in thrombocytopenia remains unclear. Possible reasons for this are as follows. Platelets fulfill vital functions in microbial host defense, angiogenesis and tissue remodeling, as well as wound healing (45–47). The higher incidence of adverse outcomes in the thrombocytopenia group can be directly explained by the decreased antimicrobial defenses of thrombocytopenia and changes in platelet function. The interaction between platelets, white blood cells, and the endothelium can result in endothelial dysfunction, leading to inflammation and thrombosis. This process is primarily mediated by platelets, which serve as the fundamental components driving this pathological cascade (7). The specific process is that platelets can be activated by microbial components or inflammatory mediators, leading to their interaction with neutrophils (48). This interaction exacerbates systemic inflammatory responses, triggers the release of inflammatory cytokines, stimulates endothelial cells, and dysregulates host defense responses by inhibiting or activating relevant signaling pathways (49). Additionally, platelets may play a chief part in the pathophysiology of disseminated intravascular coagulation (DIC) and MODS, together with the activation of endothelial cells and leukocytes (7). Although thrombocytopenia is often referred to as a condition of hypocoagulation, the reality is complex, and patients are at a potential risk for bleeding and thrombosis (50). Although different types of infection and definitions of thrombocytopenia influenced the outcome analysis, PLTs may be used to indicate infection severity.

As a suppurative infection of the liver parenchyma, PLA may be caused by a wide variety of bacteria, including biliary tract, portal vein, blood-borne or cryptogenic, and adjacent structure infections (51). Klebsiella pneumoniae was identified as the predominant bacterium in patients with PLA in our study, which aligns with findings from previous studies (52, 53). It has been reported that multibacteremia occurs in 14%–55% of PLA cases (54), a proportion slightly lower than what we observed in our study. Our study found no significant difference in bacterial culture results between the thrombocytopenia and non-thrombocytopenia groups, which was coincident with the findings of Johansson, et al. (36). However, some studies have demonstrated that thrombocytopenia is more common in gram-negative bacterial infections (55) and its duration is longer in gram-negative bacterial or fungal infections than in gram-positive bacteria (56). Unfortunately, this study lacked data on patients' platelet dynamics and did not draw similar conclusions. Owing to the differences in the distribution of microorganisms in previous studies, prevalence and duration of thrombocytopenia based on different bacterial infections remain unclear.

As mentioned above, bacterial components can directly activate platelets and release sialidase, thereby causing platelet destruction. During the deterioration of PLA, a large number of inflammatory cytokines are released, and endothelial damage occurs, triggering the activation of the coagulation cascade, which in turn result in platelet activation (7). And activated platelets lead to the production of thrombin by promoting the release of procoagulant factors, which further aggravates the development of the disease and leads to septic shock, liver and kidney function injury, and even multiple organ dysfunction in severe cases. Thrombocytopenia has been proved to be closely related to the increase of ICU occupancy rate, length of stay and mortality in critically-ill patients (57). A prospective study (58) also showed that changes in platelet mitochondrial function occurred early in patients with septic shock and were independently associated with the development of organ failure. Because thrombocytopenia induces organ damage, including renal failure, acute lung injury, and septic shock, it may indirectly lead to death (7, 57). Similarly, our findings showed that thrombocytopenia patients with PLA had nearly 7 times the risk of death, 12 times the risk of shock, and 11 times the risk of ICU transfer compared to patients without thrombocytopenia. These findings suggest that thrombocytopenia may be associated with severe infection and poor prognosis.

Additionally, previous research has indicated that the size of the abscess, gas production, and multiloculation are closely associated with the prognosis of PLA (19, 20). However, in our study, we did not observe significant differences in these variables between the two groups. Only a positive correlation between gas production and mortality was identified through univariate regression analysis. This discrepancy may be attributed to several factors, such as our relatively small sample size, missing data, confounding factors, or the heterogeneity of different ethnic groups.

In recent years, many advances have been made in the treatment of PLA. Percutaneous aspiration/drainage under US or CT guidance plus long-term antibiotic therapy has replaced traditional surgical drainage as the cornerstone of treatment (22). As in this study, most patients received antibiotics combined with puncture and drainage (60.7%). The prognosis of PLA is influenced by early diagnosis, as delayed detection can lead to complications such as liver and kidney failure, as well as respiratory failure, resulting in poor outcomes (2). However, thanks to the continuous advancements in treatment approaches, the mortality rate associated with PLA has significantly declined in recent years.

The study's limitations are as follows: (1) This is a retrospective study and lacks follow-up of patient parameters over time to directly establish absolute causality based on the results of this study. (2) Although we continuously recruited participants at our hospital and our participants were representative of local inpatients, they were not representative of the entire regional population of patients with PLA. (3) The etiological specimens were obtained from the blood or fester of patients, and different specimens for detection may have led to certain errors. (4) Data deficiencies include no details on the duration of antibiotic use, interval imaging, and a small sample size of patients reporting abscess size. (5) The data collected in this study were all indicators obtained on hospital admission without dynamic observation. In the future, a large-scale prospective study should be conducted to validate our conclusions.

## 5. Conclusion

Thrombocytopenia, an independent risk factor, was significantly associated with septic shock, ICU admission, and mortality in patients with PLA. PLT, a rapid and easy clinical laboratory index, can assist risk assessment and hierarchical management in patients with liver abscess and help identify those patients with a poor prognosis early, important for timely and appropriate intervention to improve poor outcomes. Larger sample sizes and prospective studies are required to confirm this conclusion.

## Data Availability

The original contributions presented in the study are included in the article/**Supplementary Material**, further inquiries can be directed to the corresponding authors.
