# Supplementary material for: Thrombocytopenia as an important determinant of poor prognosis in patients with pyogenic liver abscess: a retrospective case series
Source: Front Surg. 2023 Jul 25;10:1192523. doi: 10.3389/fsurg.2023.1192523 (PMC10407093; doi:10.3389/fsurg.2023.1192523)
Supplement: Supplementary file 1 [file Table1.docx]

Supplementary Material

**Thrombocytopenia as an Important Determinant of Poor Prognosis in Patients with Pyogenic Liver Abscess: A Retrospective Case Series**

**Authors:** **Shao-hua Liu ^1,2^†, Sheng-zhong Li ^3^†, Meng Hao ^4^, Tian Yu ^1,2^, Song Hu ^5^, Li Liu ^1,2*^, Zhe-long Liu ^1,2*^**

*** Correspondence:** Zhelong Liu MD PHD, Li Liu MD PHD

**E-mail**: liuzhelong@163.com (Zhelong Liu); liliu@tjh.tjmu.edu.cn (Li Liu)

| **Supplementary Table 1.** Demographics and baseline characteristics between different outcome groups with pyogenic liver abscess | | | | | | | | | |
| --- | --- | --- | --- | --- | --- | --- | --- | --- | --- |
| **Characteristics** | **Death group**  **(n=15)** | **Survival group**  **(n=443)** | **P value** | **ICU group**  **(n=14)** | **Non-ICU group**  **(n=444)** | **P value** | **Shock**  **(n=19)** | **Non-Shock**  **(n=439)** | **P** **value** |
| Age (years) | 54.7±3.1 | 52.9±0.6 | 0.602 | 50.0±3.1 | 53.1±0.6 | 0.403 | 51.3±3.4 | 53.0±0.6 | 0.574 |
| Female, n (%) | 5 (33.3) | 126 (28.4) | 0.680 | 8 (57.1) | 123 (27.7) | **0.016** | 4 (21.9) | 127  (28.9) | 0.457 |
| **Clinical symptoms/signs** |  |  |  |  |  |  |  |  |  |
| Fever, n (%) | 6 (40.0) | 113 (25.5) | 0.208 | 6 (42.9) | 113 (25.5) | 0.144 | 7 (36.8) | 112 (25.5) | 0.270 |
| Nausea and vomiting, n (%) | 2 (13.3) | 81 (18.3) | **1.000** | 3 (21.4) | 80 (18.0) | 0.726 | 5(26.3) | 78 (17.8) | 0.360 |
| Abdominal distension or pain, n (%) | 6 (40.0) | 224 (50.6) | 0.445 | 5 (35.7) | 225 (50.7) | 0.293 | 8 (42.1) | 222 (50.6) | 0.493 |
| Diarrhea, n (%) | 2 (13.3) | 34 (7.7) | 0.333 | 3 (21.4) | 33 (7.4) | 0.089 | 4 (21.1) | 32 (7.3) | 0.053 |
| Fatigue and muscle pain, n (%) | 7 (46.7) | 171 (38.6) | **0.594** | 10 (71.4) | 168 (37.8) | **0.022** | 11 (57.9) | 167 (38.0) | 0.095 |
| Chest pain, n (%) | 0 (0.0) | 18 (4.1) | 1.000 | 1 (7.1) | 17 (3.8) | 0.434 | 0 (0.0) | 18(4.1) | 1.000 |
| Palpitation, n (%) | 1 (6.7) | 26 (5.9) | **0.604** | 2 (14.3) | 25 (5.6) | 0.197 | 4 (21.1) | 23 (5.2) | **0.020** |
| Cough and sputum, n (%) | 3 (20.0) | 40 (9.0) | 0.158 | 3 (21.4) | 40 (9.0) | 0.135 | 4 (21.1) | 39 (8.9) | 0.092 |
| Dizziness or headache, n (%) | 2 (13.3) | 47 (10.6) | 0.669 | 3 (21.4) | 46 (10.4) | 0.180 | 3 (15.8) | 46 (10.5) | 0.443 |
| Dyspnea, n (%) | 1 (6.7) | 7 (1.6) | **0.235** | 4 (28.6) | 4 (0.9) | **<0.001** | 3 (15.8) | 5 (1.1) | **0.003** |
| Disturbance of consciousness, n (%) | 1 (6.7) | 6 (1.4) | 0.209 | 2 (14.3) | 5 (1.1) | **0.017** | 2 (10.5) | 5 (1.1) | **0.030** |
| **Co-morbidity** |  |  |  |  |  |  |  |  |  |
| Diabetes mellitus, n (%) | 5 (33.3) | 96 (21.7) | 0.284 | 4 (28.6) | 97 (21.8) | 0.521 | 5 (26.3) | 96 (21.9) | 0.583 |
| Hypertension, n (%) | 2(13.3) | 76 (17.2) | 0.515 | 4 (28.6) | 74 (16.7) | 0.272 | 1 (5.3) | 77 (17.5) | 0.221 |
| Cardiovascular disease, n (%) | 1 (6.7) | 12 (2.7) | 0.355 | 1 (7.1) | 12 (2.7) | 0.336 | 2 (10.5) | 11 (2.5) | 0.097 |
| Chronic respiratory disease, n (%) | 1 (6.7) | 21 (4.7) | 0.528 | 1 (7.1) | 21 (4.7) | 0.503 | 2 (10.5) | 20 (4.6) | 0.230 |
| Urologic diseases, n (%) | 0 (0.0) | 8 (1.8) | 0.765 | 0 (0.0) | 8 (1.8) | 0.779 | 0 (0.0) | 8 (1.8) | 0.711 |
| Malignancy, n (%) | 2 (13.3) | 20 (4.5) | 0.158 | 0 (0.0) | 22 (5.0) | 0.497 | 2 (9.1) | 20 (4.6) | 0.230 |
| Liver and gallbladder stones, n (%) | 4 (26.7) | 96 (21.7) | 0.750 | 4 (28.6) | 96 (21.6) | 0.517 | 5 (26.3) | 95 (21.6) | 0.579 |
| Viral hepatitis, n (%) | 1 (6.7) | 53 (12.0) | 0.454 | 3 (21.4) | 51 (11.5) | 0.221 | 2 (10.5) | 52 (11.8) | 0.607 |
| Fatty liver disease, n (%) | 2 (13.3) | 47 (10.6) | 0.669 | 2 (14.3) | 47 (10.6) | 0.653 | 1 (5.3) | 48 (10.9) | 0.708 |
| **Vital sign at admission** |  |  |  |  |  |  |  |  |  |
| Temperature, ◦C | 36.9 (36.5, 37.6) | 36.7 (36.4, 37.2) | 0.301 | 36.7 (36.0, 37.6) | 36.7 (36.4, 37.2) | 0.486 | 36.7 (36.3, 37.5) | 36.7 (36.4, 37.2) | 0.665 |
| SBP, mmHg | 115±6.9 | 120±0.9 | 0.263 | 117±4.5 | 120±0.88 | 0.451 | 104±5.5 | 121±0.85 | **<0.001** |
| DBP, mmHg | 71±3.8 | 75±0.5 | 0.117 | 72±3.8 | 75±0.6 | 0.410 | 65±3.4 | 76±0.5 | **<0.001** |
| Heart rates, /min | 95 (84, 110) | 86 (78, 98) | 0.256 | 105 (90, 117) | 86 (78, 110) | **0.007** | 95 (84, 106) | 86 (78, 98) | 0.124 |
| Respiratory rates, /min | 20 (20, 20) | 20 (20, 21) | 0.999 | 21 (20, 25) | 20 (20, 20) | 0.094 | 20 (20, 20) | 20 (20, 20) | 1.000 |
| ***Notes:*** *Data are presented as mean ±SE or median (interquartile range) for continuous variables and n (%) for categorical variables. P-values comparing thrombocytopenia group and non-thrombocytopenia group are from Student’s t-test, Mann–Whitney U-test, χ2 test, or Fisher’s exact test.*  ***Abbreviations:*** *SBP, systolic blood pressure; DBP, diastole blood pressure.* | | | | | | | | | |

| **Supplementary Table 2.** Laboratory indices between different outcome groups with pyogenic liver abscess | | | | | | | | | | |
| --- | --- | --- | --- | --- | --- | --- | --- | --- | --- | --- |
|  | **Normal range** | **Death group**  **(n=15)** | **Survival group**  **(n=443)** | **P value** | **ICU group**  **(n=14)** | **Non-ICU group**  **(n=444)** | **P value** | **Shock**  **(n=19)** | **Non-Shock**  **(n=439)** | **P** **value** |
| White blood cell, 10^9^/L | 3.5-9.5 | 10.9 (9.4, 16.3) | 10.1 (7.0, 13.7) | 0.150 | 10.9 (9.35, 21.1) | 10.1 (7.0, 13.7) | 0.513 | 9.86 (7.3, 20.1) | 10.2 (7.0, 13.6) | 0.137 |
| Neutrophil count, 10^9^/L | 1.8-6.3 | 8.8 (7.4, 14.8) | 7.8 (5.0, 11.4) | 0.055 | 8.8 (6.2, 18.1) | 7.9 (5.1, 11.4) | 0.305 | 8.8 (6.1, 16.2) | 7.8 (5.0, 11.3) | 0.093 |
| Lymphocyte count, 10^9^/L | 1.10-3.2 | 1.0 (0.6, 1.9) | 1.3 (0.9, 1.6) | 0.308 | 0.9 (0.6, 1.3) | 1.3 (0.9, 1.7) | **0.023** | 0.8 (0.5, 1.2) | 1.3 (0.9, 1.7) | **0.002** |
| Hemoglobin, g/L | 115-150 | 96 (84, 116) | 116 (102, 129) | 0.007 | 107 (83, 112) | 116 (101, 129) | **0.018** | 109 (80, 116) | 116 (101, 129) | 0.012 |
| Platelet count, 10^9^/L | 125-350 | 62 (35, 154) | 235 (145, 324) | **<0.001** | 72 (38, 239) | 235 (144, 323) | **0.002** | 48 (35, 122) | 238 (147, 326) | **<0.001** |
| CRP, mg/L | <1 | 143 (102, 173) | 115 (57, 190) | 0.560 | 189 (84, 219) | 113 (56, 185) | 0.131 | 106 (78, 173 | 116 (56, 190) | 0.723 |
| PCT, ng/ml | <0.05 | 4.07 (2.43, 19.02) | 0.93 (0.17, 7.33) | 0.105 | 1.80 (0.43, 9.31) | 0.93 (0.17, 7.11) | 0.364 | 8.86 (3.38, 40.35) | 0.80 (0.16, 6.45) | **0.003** |
| Total bilirubin, μmol/L | <=21 | 29 (11, 50) | 12 (8, 18) | **0.005** | 14 (7, 35) | 12 (8, 18) | 0.581 | 17 (7, 39) | 12 (8, 18) | 0.078 |
| Direct bilirubin, μmol/L | <=8 | 19 (6, 42) | 5 (3, 9) | **0.002** | 8 (3, 23) | 5 (3, 9) | 0.146 | 11 (3, 29) | 5 (3, 9) | **0.011** |
| ALT, U/L | <33 | 25 (15, 47) | 31 (18, 56) | 0.399 | 42 (19, 143) | 31 (18, 55) | 0.317 | 40 (17, 175) | 31 (18, 54) | 0.265 |
| AST, U/L | <32 | 31 (19, 50) | 26 (18, 45) | 0.278 | 31 (18, 126) | 26 (18, 45) | 0.307 | 30 (18, 194) | 26 (18, 45) | 0.089 |
| ALP,U/L | 135-214 | 165 (119, 301) | 139 (98, 207) | 0.068 | 154 (105, 242) | 140 (98, 210) | 0.639 | 157 (135, 301) | 139 (98, 207) | **0.049** |
| γ-GT, U/L | 6-42 | 143 (105, 167) | 115 (62, 194) | 0.424 | 77 (37, 168) | 116 (63, 194) | 0.184 | 128 (92, 157) | 115 (62, 194) | 0.670 |
| Albumin, g/L | 35-52 | 27±9 | 32±6 | 0.060 | 24±3 | 32±6 | **<0.001** | 25±6 | 32±6 | **<0.001** |
| TC, mmol/L | <5.8 | 2.2 (1.8, 3.6) | 3.1 (2.5, 3.8) | **0.011** | 2.1 (1.9, 3.2) | 3.1 (2.5, 3.8) | **0.013** | 2.0 (1.8, 3.0) | 3.2 (2.5, 3.8) | **<0.001** |
| TG, mmol/L | <1.7 | 1.4 (1.3, 10.3) | 1.2 (0.9, 1.9) | 0.120 | 1.4 (0.9, 2.7) | 1.2 (0.9, 1.8) | 0.429 | 1.3 (1.0, 1.9) | 1.2 (0.8, 1.9) | 0.595 |
| PT, s | 11.5-14.5 | 15.5 (14.3, 16.6) | 14.6 (13.9, 15.6) | 0.085 | 16.4 (15.1, 18.2) | 14.6 (13.9, 15.6) | **0.001** | 15.2 (14.3, 16.7) | 14.6, 13.9, 15.6) | 0.082 |
| APTT, s | 29-42 | 44 (39, 48) | 41 (37, 45) | 0.107 | 46 (44, 50) | 41 (37, 45) | **0.006** | 40 (36, 44) | 41 (37, 45) | 0.476 |
| Random blood glucose, mmol/L | - | 6.4 (5.5, 10.4) | 7.1 (5.6, 10.9) | 0.492 | 6.5 (6.0, 14.7) | 7.0 (5.6, 10.8) | 0.776 | 7.4 (5.8, 14.9) | 7.1 (5.6, 10.6) | 0.513 |
| BUN, mmol/L | 2.6-7.5 | 98.4 (96.5, 103.2) | 99.7 (96.3, 102.3) | 0.841 | 102.5 (97.3, 107.0) | 99.7 (96.2, 102.2) | **0.041** | 99.8 (97.3, 106.0) | 99.7 (96.2, 102.2) | 0.195 |
| Creatinine, μmol/L | 45-84 | 72 (46, 128) | 67 (54, 82) | 0.566 | 71 (56, 138) | 67 (54, 82) | 0.389 | 76 (54, 107) | 67 (54, 82) | 0.139 |
| NT-proBNP, pg/ml | <62.9 | 710 (1240, 4873) | 576 (247,2240) | 0.102 | 660 (408, 12293) | 683 (199, 2268) | 0.290 | 2212 (764, 12293) | 474 (199, 1681) | **0.001** |
| cTnI , pg/mL | <=34.2 | 0.91 (0.01, 72.15) | 1.81 (0.01,7.63) | 0.891 | 2.20 (0.02, 7.75) | 1.05 (0.01, 7.20) | 0.549 | 3.50 (0.15, 79.95) | 1.05 (0.00, 6.15) | 0.078 |
| ***Notes:*** *Data are presented as median (interquartile range) for continuous variables. P-values comparing thrombocytopenia and non-thrombocytopenia group are from Mann–Whitney U-test.*  ***Abbreviations:*** *CRP, C-reactive protein; PCT, procalcitonin; ALT, alanine aminotransferase; AST, aspartate aminotransferase, ALP, alkaline phosphatase; γ-GT, γ-glutamate transpeptidase；TC, Total cholesterol；TG, Triglyceride; HDL-C, high-density lipoprotein cholesterol; LDL-C, low-density lipoprotein cholesterol; PT, prothrombin time; APTT, activated partial thromboplastin time; Fib, Fibrinogen; BUN, blood urea nitrogen；NT-proBNP, n-terminal pro-brain natriuretic peptide; cTnI, cardiac troponin I.* | | | | | | | | | | |

| **Supplementary Table 3.** Treatment and clinical outcomes between different outcome groups with pyogenic liver abscess | | | | | | | | | |
| --- | --- | --- | --- | --- | --- | --- | --- | --- | --- |
|  | **Death group (n=15)** | **Survival group**  **(n=443)** | **P value** | **ICU group**  **(n=14)** | **Non-ICU group**  **(n=444)** | **P value** | **Shock**  **(n=19)** | **Non-Shock**  **(n=439)** | **P** **value** |
| **Treatments** |  |  |  |  |  |  |  |  |  |
| Antibiotics alone | 7 (46.7) | 121 (27.3) | 0.100 | 2 (14.3) | 126 (28.4) | 0.368 | 5 (26.3) | 123 (28.0) | 0.871 |
| Antibiotics plus percutaneous drainage | 6 (40.0) | 272 (61.4) | 0.095 | 10 (71.4) | 268 (60.4) | 0.580 | 12 (63.2) | 266 (60.6) | 0.823 |
| Antibiotics plus surgical | 0 (0.0) | 33 (100) | 0.615 | 2 (14.3) | 31 (7.0) | 0.267 | 1 (5.3) | 32 (7.3) | 0.100 |
| Albumin infusion | 12 (80) | 191 (43.1) | **0.018** | 13 (92.9) | 190 (42.8) | **0.001** | 14 (73.7) | 189 (43.1) | **0.031** |
| Antiviral drug | 1 (6.7) | 28 (63) | 0.993 | 3 (21.4) | 26 (5.9) | 0.06 | 2 (10.5) | 27 (6.2) | 0.688 |
| **Serious complications** |  |  |  |  |  |  |  |  |  |
| Septic shock | 8 (53.3) | 11 (2.5) | **<0.001** | 6 (42.9) | 13 (2.9) | **<0.001** | - | - | - |
| Acute renal injury | 3 (20.0) | 25 (5.6) | **0.007** | 3 (21.4) | 25 (5.6) | **0.049** | 4 (21.1) | 24 (5.5) | **0.020** |
| Acute hepatic injury | 12 (80.0) | 200 (45.1) | **0.009** | 9 (64.3) | 203 (45.7) | 0.170 | 15 (78.9) | 197 (44.9) | **0.004** |
| Heart failure | 4 (26.7) | 26 (5.9) | **0.012** | 5 (35.7) | 25 (5.6) | **<0.001** | 9 (47.4) | 21 (4.8) | **<0.001** |
| Myocardial infarction | 3 (20.0) | 10 (2.3) | **0.007** | 3 (21.4) | 10 (2.3) | **0.005** | 6 (31.6) | 7 (1.6) | **<0.001** |
| Pulmonary edema | 4 (26.7) | 8 (1.8) | **<0.001** | 6 (42.9) | 6 (1.4) | **<0.001** | 8 (42.1) | 4 (0.9) | **<0.001** |
| ARDS | 4 (26.7) | 6 (1.4) | **<0.001** | 6 (42.9) | 4 (0.9) | **<0.001** | 7 (36.8) | 3 (0.7) | **<0.001** |
| Pleural effusion | 9 (60.0) | 178 (40.2) | 0.125 | 12 (85.7) | 175 (39.4) | **0.001** | 13 (68.4) | 174 (39.6) | **0.012** |
| **ICU admission** | 4 (26.7) | 10 (2.3) | **0.001** | - | - | - | 6 (31.6) | 8 (1.8) | **<0.001** |
| **Mortality** | - | - | - | 4 (28.6) | 11 (2.5) | **0.001** | 8 (42.1) | 7 (1.6) | **<0.001** |
| ***Notes:*** *Data are presented as n (%) for categorical variables. P-values comparing thrombocytopenia and non-thrombocytopenia group are from χ2 test or Fisher's exact test.*  ***Abbreviations:*** *ARDS, acute respiratory distress syndrome.* | | | | | | | | | |
