# Supplementary figures and images for: Thrombocytopenia as an important determinant of poor prognosis in patients with pyogenic liver abscess: a retrospective case series
Source: Front Surg. 2023 Jul 25;10:1192523. doi: 10.3389/fsurg.2023.1192523 (PMC10407093; doi:10.3389/fsurg.2023.1192523)

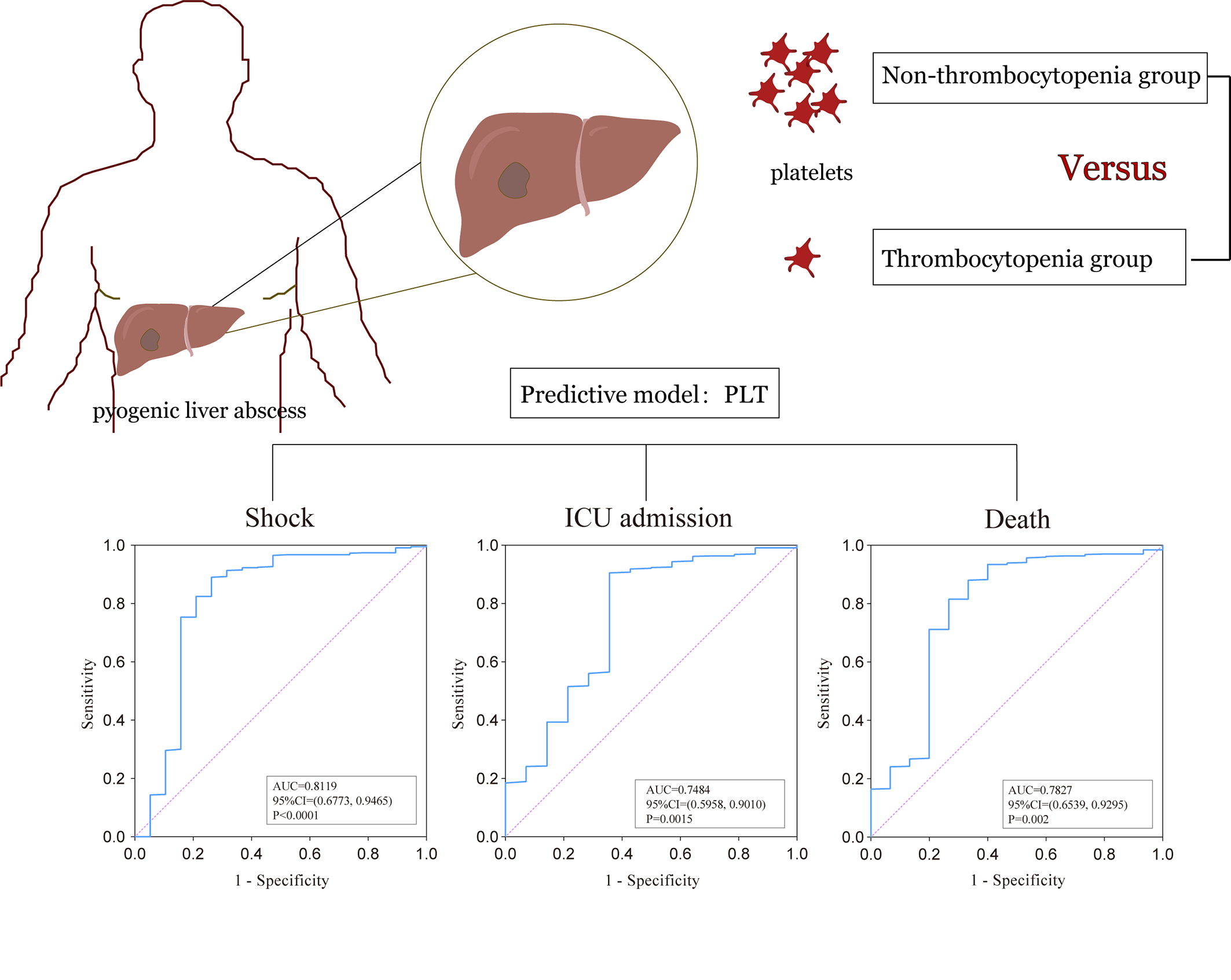

Supplement: Supplementary file 2 [file Image1.tif]
